# Differential expression of Hsp27 in normal oesophagus, Barrett's metaplasia and oesophageal adenocarcinomas

**DOI:** 10.1038/sj.bjc.6690094

**Published:** 1999-02

**Authors:** O S Soldes, R D Kuick, I A Thompson II, S J Hughes, M B Orringer, M D Iannettoni, S M Hanash, D G Beer

**Affiliations:** Thoracic Tumor Biology Laboratory, Section of Thoracic Surgery, Department of Surgery, University of Michigan Medical Center, Ann Arbor, MI 48109, USA; Department of Pediatrics, University of Michigan Medical Center, Ann Arbor, MI 48109, USA

**Keywords:** heat shock protein 27, Barrett's metaplasia, oesophagus, cancer, adenocarcinoma

## Abstract

The protein expression patterns of normal, metaplastic and malignant oesophageal tissues were analysed by two-dimensional polyacrylamide gel electrophoresis (2D-PAGE) to identify changes associated with Barrett's metaplasia and transformation to oesophageal adenocarcinoma. Heat-shock protein 27 (Hsp27), a small heat-shock protein which is protective against cytotoxic stresses, was abundant in normal oesophagus. However, Hsp27 expression was markedly lower in Barrett's metaplasia and oesophageal adenocarcinomas. This was confirmed by immunohistochemical analysis. Hsp27 protein was most highly expressed in the upper layers of squamous epithelium and exhibited a pattern of expression that corresponded with the degree of squamous maturation. Northern and Southern analysis demonstrated Hsp27 to be regulated at the level of mRNA transcription or abundance. Normal oesophageal tissues were examined for gender differences in Hsp27 expression. Women expressed fourfold higher levels of Hsp27 mRNA, however, this difference was not appreciable in protein expression. Hsp27 protein was inducible by heat shock in Barrett's adenocarcinoma cell lines and an immortalized oesophageal epithelial cell line (HET-1A), but not by oestradiol. These results demonstrate abundant constitutive expression of the stress-response protein Hsp27 in the normal oesophagus, and suggest that low-level expression in Barrett's metaplasia may be one factor which may influence susceptibility to oesophageal adenocarcinoma development. © 1999 Cancer Research Campaign


*British Journal of Cancer* (1999) **79**(3/4), 595–603
© 1999 Cancer Research Campaign
Article no. bjoc.1998.0094

### Differential expression of Hsp27 in normal oesophagus, Barrett's metaplasia and oesophageal adenocarcinomas

**OS Soldes[1], RD Kuick[2], IA Thompson II[1], SJ Hughes[1], MB Orringer[1], MD Iannettoni[1], SM Hanash[2] and DG Beer[1]**

[1]Thoracic Tumor Biology Laboratory, Section of Thoracic Surgery, Department of Surgery, and [2]Department of Pediatrics, University of Michigan Medical Center, Ann Arbor, MI 48109, USA

**Summary** The protein expression patterns of normal, metaplastic and malignant oesophageal tissues were analysed by two-dimensional polyacrylamide gel electrophoresis (2D-PAGE) to identify changes associated with Barrett's metaplasia and transformation to oesophageal adenocarcinoma. Heat-shock protein 27 (Hsp27), a small heat-shock protein which is protective against cytotoxic stresses, was abundant in normal oesophagus. However, Hsp27 expression was markedly lower in Barrett's metaplasia and oesophageal adenocarcinomas. This was confirmed by immunohistochemical analysis. Hsp27 protein was most highly expressed in the upper layers of squamous epithelium and exhibited a pattern of expression that corresponded with the degree of squamous maturation. Northern and Southern analysis demonstrated Hsp27 to be regulated at the level of mRNA transcription or abundance. Normal oesophageal tissues were examined for gender differences in Hsp27 expression. Women expressed fourfold higher levels of Hsp27 mRNA, however, this difference was not appreciable in protein expression. Hsp27 protein was inducible by heat shock in Barrett's adenocarcinoma cell lines and an immortalized oesophageal epithelial cell line (HET-1A), but not by oestradiol. These results demonstrate abundant constitutive expression of the stress-response protein Hsp27 in the normal oesophagus, and suggest that low-level expression in Barrett's metaplasia may be one factor which may influence susceptibility to oesophageal adenocarcinoma development.

**Keywords:** heat shock protein 27; Barrett's metaplasia; oesophagus; cancer; adenocarcinoma

The incidence of oesophageal adenocarcinoma has been rising in the United States and other Western nations (Hesketh et al, 1989; Blot et al, 1991, Reed and Johnson, 1993). This has stimulated interest in the biology of the oesophageal mucosa and Barrett's metaplasia and the factors that may predispose to the development of oesophageal adenocarcinoma. Barrett's oesophageal metaplasia is a premalignant lesion in which the normal squamous mucosa of the distal oesophagus is replaced by columnar epithelium (Barrett, 1950; Barrett, 1957). It is associated with a 30- to 40-fold increase in the risk of oesophageal adenocarcinoma (Spechler et al, 1984; Van Der Veen et al, 1989), which develops in approximately 10% of patients with Barrett's metaplasia (Naef et al, 1975). Barrett's metaplasia is commonly thought to arise as a consequence of chronic gastro-oesophageal reflux-induced injury (Goldman and Beckman, 1960; Bremner et al, 1970). The prevalence of Barrett's metaplasia and oesophageal adenocarcinoma is higher in men than in women and is highest in Caucasians (Skinner et al, 1983; Cameron et al, 1985; Reed and Johnson, 1993). Clinically, Barrett's metaplasia is found in approximately 12% of patients who undergo oesophagogastroduodenoscopy for chronic reflux symptoms (Schnell et al, 1985; Winters et al, 1987).

The human oesophagus is normally lined by a layer of protective non-keratinizing stratified squamous epithelium, which is adapted to withstand physical stresses such as abrasion (Wheater et al, 1987). Squamous tissues in other sites (e.g. skin and parabasal cell layer of squamous cervical epithelium) have been found to express the 27-kDa stress-response peptide, Hsp27 (Dressler et al, 1986; Trautinger et al, 1995). Hsp27 belongs to the family of stress response or heat-shock proteins. These proteins are synthesized in response to diverse cytotoxic stresses and produce a transient increase in tolerance to subsequent injury. Increased expression of Hsp27 protein has been demonstrated to protect against a variety of toxic stresses including heat shock, oxidative injury, heavy metals, antineoplastic agents and tumour necrosis factor α (TNF-α) (Landry et al, 1989, Mehlen et al, 1995*a*, 1995*b*; Hout et al, 1991, 1996; Richards et al, 1996; Wu and Welsh, 1996). Hsp27 is a cytoplasmic protein that is constitutively expressed in a broad range of normal tissues and neoplasms. However, wide variations in the degree of expression are observed between different cell types and tissues (Ciocca et al, 1983; Dunn et al, 1993).

Hsp27 is a small phosphoprotein with multiple isoforms. There is an unphosphorylated isoform (Hsp27A), two major phosphoisoforms (Hsp27B and Hsp27C) (Arrigo et al, 1987) and a minor phosphoisoform (Hsp27D) (Welch 1985; Landry et al, 1992). Hsp27 phosphorylation is necessary for the proper protective functioning of the protein (Lavoie et al, 1995; Huot et al, 1996). In unstressed cells, the predominant isoform is Hsp27A. Exposure to heat shock and other stresses causes synthesis and accumulation of Hsp27 over several hours, whereas redistribution of the protein to the phosphorylated isoforms occurs within minutes (Landry et al, 1991, 1992).

In this study, we analysed the polypeptide pattern of normal, metaplastic and malignant oesophageal tissues by two-dimensional polyacrylamide gel electrophoresis (2D-PAGE) to identify changes in protein expression associated with Barrett's metaplasia

*Received 17 February 1998*
*Revised 24 April 1998*
*Accepted 6 May 1998*

*Correspondence to*: DG Beer, Thoracic Tumor Biology Laboratory, MSRB II B560, Box 0686, University of Michigan, 1150 W Medical Center Drive, Ann Arbor, MI 48109, USA

and transformation to oesophageal adenocarcinoma. Two polypeptides were noted to be highly abundant in the normal oesophagus and consistent with Hsp27 phosphoisoforms we previously identified by 2D-PAGE and sequenced (Hanash et al, 1986, 1988; Strahler et al, 1990). We observed that Hsp27 is abundantly expressed in the normal oesophageal mucosa and exhibits a pattern of protein expression that corresponds with the degree of squamous cell maturation. However, Barrett's metaplasia and Barrett's adenocarcinomas express significantly lower levels of Hsp27 mRNA and protein. Women patients express fourfold higher levels of Hsp27 mRNA in their oesophagus than men, but comparable differences in the level of Hsp27 protein were not observed. Because Hsp27 was first identified in breast tumours (MCF-7) as an oestrogen-responsive protein (Edwards et al, 1980), we examined the response of adenocarcinoma cell lines and an oesophageal epithelial cell line (HET-1A) to both heat shock and oestradiol treatment. Hsp27 protein was inducible by heat shock in Barrett's adenocarcinoma cell lines and in HET-1A, but not by oestradiol.

### MATERIALS AND METHODS

### Patients and tissues

After informed consent, tissues were obtained from the surgical specimens of 56 patients undergoing oesophagectomy for malignancy or high-grade dysplasia at the University of Michigan Medical Center (Ann Arbor, MI, USA). Thirty-five patients were men (mean age 66 years; range 45–84 years) and 21 were women (mean age 68 years; range 52–88 years). Eighteen patients had Barrett's metaplasia and oesophageal adenocarcinoma (Barrett's adenocarcinoma), two had Barrett's metaplasia with high-grade dysplasia, five patients had adenocarcinomas of the distal oesophagus/gastric cardia without Barrett's metaplasia (cardia tumours), and 24 patients had squamous cell carcinomas. Most patients had advanced stage tumours at the time of oesophagectomy (stage III or IV). Tissues were placed in Dulbecco's modified Eagle medium (DMEM) (Gibco, Grand Island, NY, USA) on ice immediately after resection. Surgical specimens for cryostat sectioning were trimmed of connective tissue and muscularis propria, embedded in OCT compound (Miles Scientific, Naperville, IL, USA) and frozen in isopentane cooled to the temperature of liquid nitrogen. Additional tissue was frozen in liquid nitrogen for DNA, RNA, or protein isolation and stored at –70°C until analysed. An oesophageal cancer database and patient charts were reviewed for clinical information.

### Cell lines

Three oesophageal Barrett's adenocarcinoma cell lines (Seg-1, Flo-1, Bic-1) and an oesophageal epithelial cell line (HET-1A) were also used in this study. The characterization of the oesophageal adenocarcinoma cell lines is described elsewhere (manuscript in preparation). HET-1A is a human epithelial cell line immortalized by transfection of the SV40 T antigen early region gene (Stoner et al, 1991). All four cell lines were cultured in DMEM supplemented with 10% fetal bovine serum (FBS) and 100 U $ml^{-1}$ penicillin G and 100 μg $ml^{-1}$ streptomycin, at 37°C in 5% carbon dioxide/95% air. Culture dishes (Corning Glass Works, Corning, NY, USA) for HET-1A culture were pretreated by coating the plates with heat-inactivated FBS or 100 μg $ml^{-1}$ bovine serum albumin (BSA) (Sigma, St. Louis, MO, USA) and 20 μg $ml^{-1}$ type I rat tail collagen (Beckton Dickinson Labware, Bedford, MA, USA) and incubated at 37°C for 1–2 h.

### Quantitative 2D-PAGE

Cell lines and human tissues were solubilized in lysis buffer composed of 9.5 M urea, 2% NP-40, 2% ampholines (pH 3.5–10; Pharmacia/LKB, Piscataway, NJ, USA), 2% 2-mercaptoethanol and 0.2 mM phenylmethylsulphonylfluoride. Samples were isoelectrically focused in the first dimension at 700 V for 16 h followed by 1000 V for 2 h at ambient temperature. Second-dimension separation by size was in 11–14% polyacrylamide gradient-SDS gels. The gels were silver stained and digitized for computer quantitation of Hsp27 protein spots (Kuick et al, 1987). The Hsp27 isoforms were identified on the basis of their characteristic migration and unique position in relation to the constellation of neighbouring landmark spots. The integrated intensities of the Hsp27 isoform spots Hsp27A (unphosphorylated), the major phosphorylated isoform Hsp27B and ten other reference spots were determined (optical density × $mm^2$). The phosphorylated isoform Hsp27C was difficult to identify reliably and quantitate in many specimens, because of either small spot size or proximity to other protein spots on the stained gels, and was not quantitated and analysed. Reference spots were used to adjust the Hsp27 spots' integrated intensity to compensate for any variability in the amount of protein loaded or in gel staining. Raw spot integrated intensities are divided by an adjustment factor based on reference spot intensity to obtain adjusted integrated intensities. A Student *t*-test was used to compare spot intensities between groups of tissues. A *P*-value of 0.05 was deemed significant.

### Immunohistochemistry

Sections of tissues (5 μm) were mounted on 0.01% poly-L-lysine-coated slides and fixed in acetone at –20°C for 10 min. Endogenous peroxidase activity was quenched using two changes of 0.5% hydrogen peroxide in PBS for 30 min each. After a blocking step with a 1:20 dilution of horse serum, the tissue sections were incubated with a mouse monoclonal antibody to Hsp27 (Clone G3.1, Lab Vision/NeoMarkers, Fremont, CA, USA) at a 1:100 dilution in 1% BSA–PBS. PBS was used in place of the primary antibody on negative control sections stained simultaneously with all test sections. Immunoreactivity was detected with a Vectastain detection kit (Vectastain ABC Mouse IgG kit, Vector Laboratories, Burlingame, CA, USA) and diaminobenzidine was used as a chromogen. The slides were lightly counterstained with Harris modified haematoxylin (Fisher Scientific, Fairlawn, NJ, USA) and coverslips mounted. The pattern and intensity of staining were evaluated by two examiners using light microscopy. The intensity of Hsp27 staining in the epithelia or tumour was classified as high, low or negative for each tissue.

### Heat shock treatment of cell lines

Cell lines were grown to 50–90% confluence in 100-mm sterile tissue culture dishes at 37°C. Fresh 37°C medium was added and the cultures were incubated at 42°C for 2 h, and then returned to the 37°C incubator for 16 or 24 h until harvesting. The controls for each cell line were incubated at 37°C for the same total time. After incubation, the cells were collected with a rubber policeman, pelleted and frozen at –70°C.

© Cancer Research Campaign 1999

**Table 1** 2D-PAGE analysis of normal oesophagus, Barrett's metaplasia, Barrett's adenocarcinoma, cardia tumours, gastric tissue and squamous cell carcinoma to quantify Hsp27 protein isoforms

| Tissue type (*n*) | Mean Hsp27A | Mean Hsp27B |
|---|---|---|
| Normal oesophagus (23) | 4.6 ± 0.3 | 4.1 ± 0.2 |
| Barrett's metaplasia (10) | 1.2 ± 0.3[a] | 1.2 ± 0.1[a] |
| Barrett's adenocarcinoma (11) | 1.0 ± 0.1[a] | 0.9 ± 0.1[a] |
| Cardia tumours (5) | 0.9 ± 0.1[a] | 1.0 ± 0.1[a] |
| Gastric (5) | 1.1 ± 0.3[a] | 0.9 ± 0.2[a] |
| Squamous cell carcinoma (6) | 1.8 ± 0.4[a] | 1.8 ± 0.4[a] |

[a]Significantly different from normal oesophagus ($P < 0.001$), using Student's *t*-test.

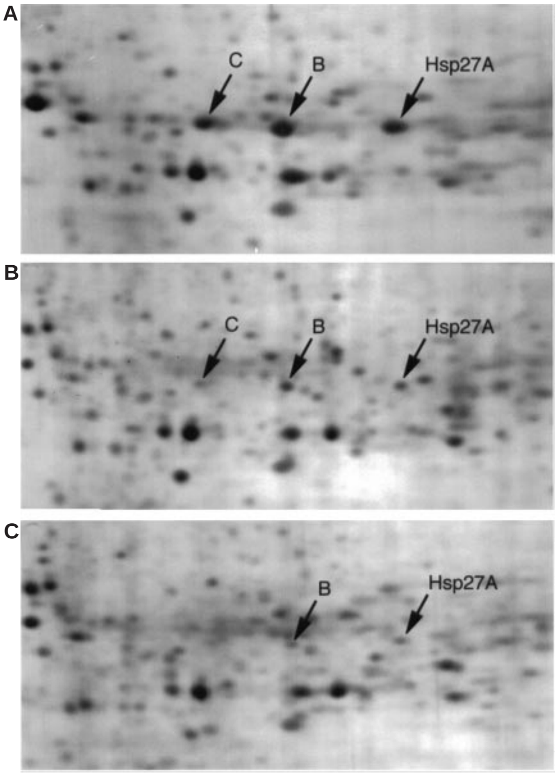

**Figure 1** 2D-PAGE polypeptide patterns of (**A**) normal oesophagus, (**B**) Barrett's metaplasia and (**C**) Barrett's adenocarcinoma tissue specimens from the same patient. The Hsp27A and B isoform spots are markedly smaller in Barrett's metaplasia and Barrett's adenocarcinomas than in normal oesophagus. The Hsp27C isoform is difficult to detect in the Barrett's adenocarcinoma (C)

### Oestradiol treatment of cell lines

Fresh media (10 ml) and 10 μl of 0.01, 0.1 or 1 mM 17β-oestradiol in 100% ethanol were added to cultures of HET-1A and Seg-1 (80% confluent) to achieve oestradiol concentrations of 10, 100 or 1000 nM. Ten microlitres of 100% ethanol was added to control cultures. The dishes were incubated for 48 h at 37°C and the cells were collected as described above.

### DNA constructs and probes

The human Hsp27 cDNA insert of the plasmid pHS 208 (Hickey et al, 1986) was radiolabelled with [$^{32}$P]dCTP using a Random Primers DNA Labeling System (Life Technologies, Gaithersburg, MD, USA) and used as a probe for Northern and Southern blot analysis. A glutathione-S-transferase pi (GSTπ) cDNA probe was made according to a nucleotide sequence derived from the human glutathione-S-transferase pi cDNA (Moscow et al, 1989). Oligonucleotide primers (sense = 5′-CTCAAAGCCTCCTGCC-TATAC-3′ and anti-sense = 5′-GGTAGTTACCGTTGCCCTTTG-3′) were designed and reverse transcriptase polymerase chain reaction (RT-PCR) was performed to amplify GSTπ using the total RNA isolated from normal squamous oesophagus. Total RNA (2 μg) was reverse transcribed using random hexamer primers (Promega, Madison, WI, USA) and avian myeloblast RT (Boehringer Mannheim, Indianapolis, IN, USA). PCR of GSTπ was performed by adding 16 μl of RT mixture to a 100-μl reaction of 10 mmol $l^{-1}$ tris-HCl (pH 8.3), 50 mmol $l^{-1}$ potassium chloride, 1.5 mmol $l^{-1}$ magnesium chloride, 2 mmol $l^{-1}$ dithiothreitol (DTT), 0.5 mmol $l^{-1}$ deoxynucleotide triphosphate (dNTPs) (100 mM), 12.5 pmol of each GST primer, and 0.5 μl *Taq* polymerase (Promega). Amplification was performed with an initial cycle of 94°C for 1 min followed by 35 cycles of: 94°C for 1 min, 57°C for 2 min, and 72°C for 3 min. The amplified GSTπ cDNA probe was purified and radiolabelled with [$^{32}$P]dCTP using the Random Primers DNA Labeling System.

### Northern analysis

Total RNA was isolated from cell lines or tissues using Trizol Reagent (Gibco) according to the manufacturer's instructions. For each sample, 10 μg of RNA was used to prepare Northern blots which were hybridized with the radiolabelled Hsp27 probe, washed and exposed to autoradiography film (Hyperfilm-MP, Amersham International, Buckinghamshire, UK) as previously described (Rachwal et al, 1995). To correct for any variability in loading and transfer, the RNA signals were normalized using a probe for 28S rRNA (Hanson et al, 1991). The RNA signals on the film were measured by laser densitometry (Molecular Dynamics, Sunnyvale, CA, USA).

### Southern analysis

DNA was isolated from the frozen tissues and cell pellets by proteinase K digestion, serial phenol extraction and ethanol precipitation using standard procedures. For each sample, 10 μg DNA was digested overnight at 37°C with *Eco*RI restriction enzyme (Promega, Madison, WI, USA) and Southern blots prepared as previously described (Al-Kasspooles et al, 1993). The blotted membranes were hybridized with the $^{32}$P-radiolabelled human Hsp27 cDNA probe, washed and autoradiographs prepared.

### Western blotting

Protein for Western blotting was isolated from tissues from the phenol–ethanol supernatant of Trizol Reagent (Gibco) according to the manufacturer's instructions. Samples (10 μg) of protein were size fractionated using 15% polyacrylamide mini-gels and transferred onto polyvinylidene difluoride membranes (Immobilon-P, Millipore, Bedford, MA, USA). Membranes were blocked in 3% non-fat milk in Tris-buffered saline (TBS). Blotted membranes were probed with the G3.1 clone anti-Hsp27 antibody at a 1:500 or 1:1000 dilution in TBST (TBS with 0.1% Tween-20), and then

© Cancer Research Campaign 1999

**Table 2** Hsp27 protein expression examined by immunohistochemistry

| | | Staining intensity | | |
|---|---|---|---|---|
| **Tissue** | ***n*** | **High** | **Low** | **Negative** |
| Normal oesophagus | 27 | 27 | 0 | 0 |
| Barrett's metaplasia | 13 | 3 | 4 | 6 |
| Barrett's adenocarcinoma | 13 | 3 | 2 | 8 |
| Cardia tumours | 5 | 0 | 0 | 5 |
| Gastric | 3 | 0 | 0 | 3 |
| Squamous cell carcinoma | 7 | 7 | 0 | 0 |

incubated with goat anti-mouse horseradish peroxidase-conjugated antibody (Chemicon International, Temecula, CA, USA), diluted 1:2500 in TBST. Primary antibody binding was detected with an enhanced chemiluminescence kit (Pierce, Rockford, IL, USA).

### RESULTS

### Hsp27 levels in normal oesophageal tissue, Barrett's metaplasia and oesophageal carcinomas

Tissue samples of normal oesophagus, Barrett's metaplasia, Barrett's adenocarcinoma and squamous cell oesophageal carcinoma from 23 patients were analysed by 2D-PAGE. Barrett's metaplasia samples were not available for analysis in one patient with Barrett's adenocarcinoma. Another patient's tumour had an adenosquamous histology, and this tumour was excluded from the analysis. The normal oesophagus samples demonstrated a high mean integrated spot intensity of both unphosphorylated (Hsp27A) and phosphorylated (Hsp27B) isoforms of Hsp27. The samples of Barrett's metaplasia and Barrett's adenocarcinoma had significantly lower mean integrated spot intensities of both Hsp27A and B than normal oesophagus (Table 1 and Figure 1). Tumours of the cardia and gastric tissue from five patients demonstrated similar

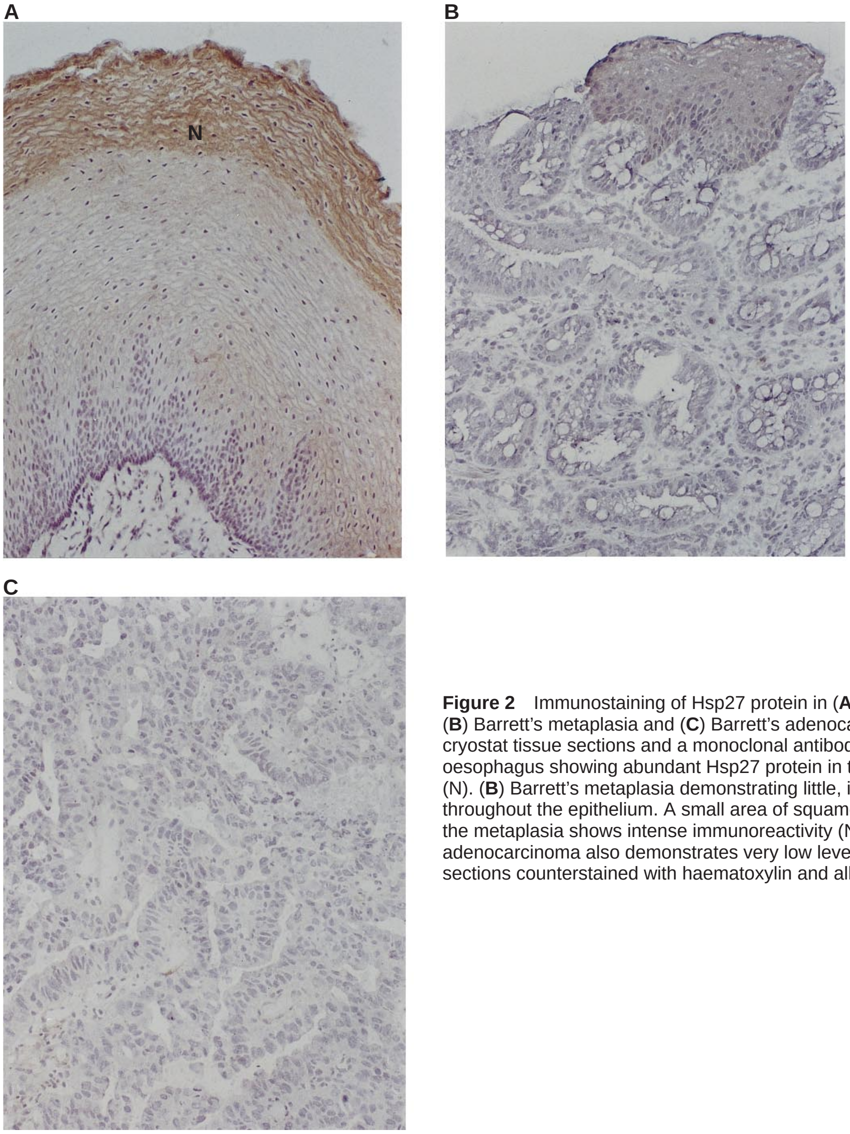

**Figure 2** Immunostaining of Hsp27 protein in (**A**) normal oesophagus, (**B**) Barrett's metaplasia and (**C**) Barrett's adenocarcinoma, using 5-µm cryostat tissue sections and a monoclonal antibody to Hsp27. (**A**) Normal oesophagus showing abundant Hsp27 protein in the squamous epithelium (N). (**B**) Barrett's metaplasia demonstrating little, if any, immunoreactivity throughout the epithelium. A small area of squamous epithelium adjacent to the metaplasia shows intense immunoreactivity (N). (**C**) Barrett's adenocarcinoma also demonstrates very low levels of Hsp27 expression. All sections counterstained with haematoxylin and all magnifications ×70

 *© Cancer Research Campaign 1999*

**Table 3** Northern analysis of normal oesophagus, Barrett's metaplasia, Barrett's adenocarcinoma, and gastric tissue hybridized with a $^{32}$P-labelled Hsp27 cDNA and then rehybridized with a $^{32}$P-labelled 28S rRNA probe to normalize for RNA loading and transfer. The relative mean Hsp27/28S mRNA signals are shown, with the mean level in normal oesophagus set at 100

| Sample | *n* | Mean Hsp27/28S mRNA |
|---|---|---|
| Normal oesophagus | 7 | 100 |
| Barrett's metaplasia | 6 | 13 |
| Barrett's adenocarcinoma | 7 | 30 |
| Gastric | 2 | 4 |

findings of low levels of both isoforms A and B. Squamous cell cancers also had lower mean integrated spot intensities of both isoforms than the normal oesophageal tissues. However, the mean spot intensities of the squamous cell tumours were higher than the mean spot intensities of either the adenocarcinomas or Barrett's metaplasia. The Hsp27 levels in the tissue sample groups were compared with the levels in normal squamous oesophagus. Unpaired two-tailed Student's *t*-tests revealed differences between normal oesophagus and other tissue sample groups to be statistically significant ($P < 0.001$) (Table 1).

The 2D-PAGE findings were confirmed by Hsp27 immunohistochemical analysis of oesophageal tissues from each of 27 patients (Table 2). Normal oesophagus was compared with other tissues in the same patients. There was abundant expression of Hsp27 protein in the cytoplasm of cells comprising the stratified squamous epithelium of the oesophagus in a pattern similar to the human epidermis (Trautinger et al, 1995). Hsp27 protein staining was lowest at the basal (regenerative) layer, gradually increasing to intense staining in the post-mitotic cells of the upper functional layers close to the luminal surface. The intensity of cytoplasmic staining correlates with the degree of maturity of the epithelial cells (Figure 2). The muscularis mucosae also demonstrated strong staining for Hsp27, but the oesophageal submucosal glands were negative. The majority of the Barrett's metaplasia (10 out of 13) and Barrett's adenocarcinoma (10 out of 13) sections were graded as having either low or negative staining of the metaplastic epithelium and tumour cells (Figure 2B and C), with only a few samples showing high-intensity cytoplasmic staining. All of the cardia tumours and the gastric tubular mucus-secreting glands were negative for Hsp27 staining. In contrast, the seven squamous cell oesophageal carcinomas all displayed high-intensity cytoplasmic staining.

### Hsp27 expression is transcriptionally regulated

Oesophageal and gastric tissues from seven patients were examined by Northern blot analysis. In all cases, normal squamous oesophagus had significantly higher levels of Hsp27 mRNA than the Barrett's metaplasia, Barrett's adenocarcinoma, or gastric tissue (Figure 3A and B). Gastric tissue demonstrated very low levels of Hsp27 mRNA. The mean normalized mRNA levels (Hsp27/28S RNA ratios) in the tissue types are shown in Table 3.

Hsp27 mRNA levels were also determined for normal oesophagus and squamous cell oesophageal carcinoma from five patients. The mean normalized Hsp27 mRNA levels in the tumours were slightly lower than in normal oesophagus (5.2 vs. 4.9), but this difference was not statistically significant. However, the levels present in squamous cell carcinomas are much higher than in either Barrett's metaplasia or Barrett's adenocarcinomas.

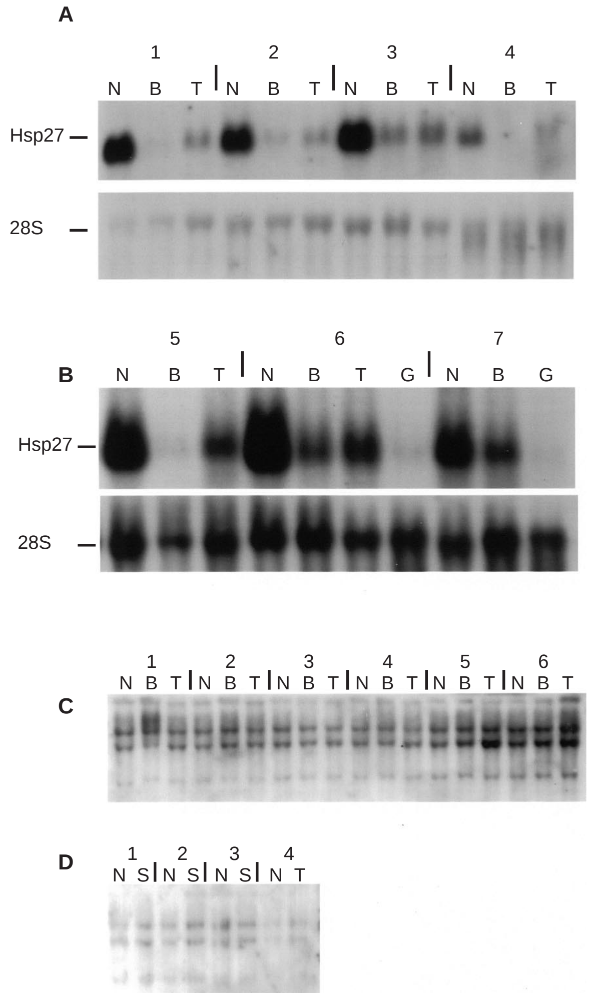

**Figure 3** (**A, B**) Northern analysis of Hsp27 mRNA expression. Northern blots of RNA from samples of normal oesophagus (N), Barrett's metaplasia (B), Barrett's adenocarcinoma (T) and gastric tissue (G) were hybridized with a $^{32}$P-labelled Hsp27 cDNA. Normal squamous oesophagus shows markedly higher expression of Hsp27 mRNA than Barrett's metaplasia or adenocarcinoma from the same patients (cases 1–6). The Barrett's adenocarcinomas express more Hsp27 mRNA than the Barrett's metaplasia. (**B**) Gastric tissue (G) also expresses very low levels of Hsp27 mRNA (cases 6 and 7). (**C, D**) Southern analysis of Hsp27 in *Eco*RI-digested DNA samples from normal oesophagus, Barrett's metaplasia, Barrett's adenocarcinomas and oesophageal squamous cell carcinomas (S). (**C**) No major deletions or gross alteration of the Hsp27 gene was apparent in the Barrett's metaplasias or adenocarcinomas (cases 1–6) or (**D**) in the squamous cell carcinomas (cases 1–3). Hybridization with the Hsp27 cDNA results in three specific Hsp27-gene bands with *Eco*RI digest. Case 4 is the normal oesophagus and tumour from a patient with Barrett's adenocarcinoma

Southern blot analysis was performed to rule out genomic loss or major structural changes in the Hsp27 gene as a basis for reduced expression. The normal oesophagus, Barrett's metaplasia and tumours from seven patients with Barrett's adenocarcinomas, and the normal oesophagus and tumours of three patients with squamous cell carcinomas were examined. No evidence of deletion or gross alteration of the gene was observed in any tissues (Figure 3C and D).

### Hsp27 expression in relation to gender

Given the higher incidence of oesophageal adenocarcinoma in men, potential gender-related differences in Hsp27 expression

© Cancer Research Campaign 1999

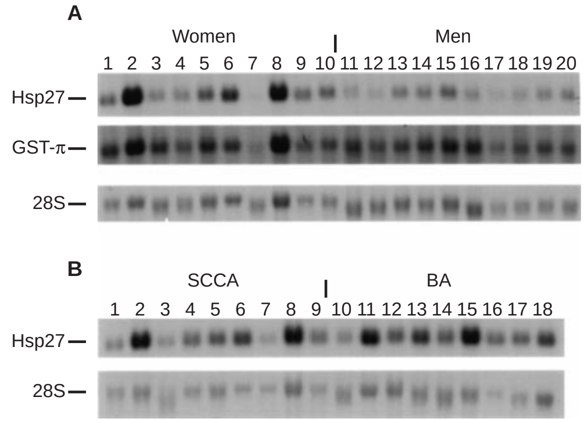

**Figure 4** Hsp27 mRNA expression in the normal oesophagus of women and men. (**A**) A Northern blot was prepared from the normal oesophagus of ten women (lanes 1–10) and ten men (lanes 11–20) with the diagnosis of squamous cell carcinoma of the oesophagus and hybridized with a $^{32}P$-labelled Hsp27 cDNA. The blot was stripped and sequentially rehybridized with a $^{32}P$-labelled 28S rRNA oligomer and a $^{32}P$-labelled GSTπ cDNA probe. (**B**) Northern analysis of Hsp27 mRNA in the normal oesophagus of nine women with squamous cell carcinoma (SCCA, lanes 1–9) and nine women with Barrett's adenocarcinomas (BA, lanes 10–18)

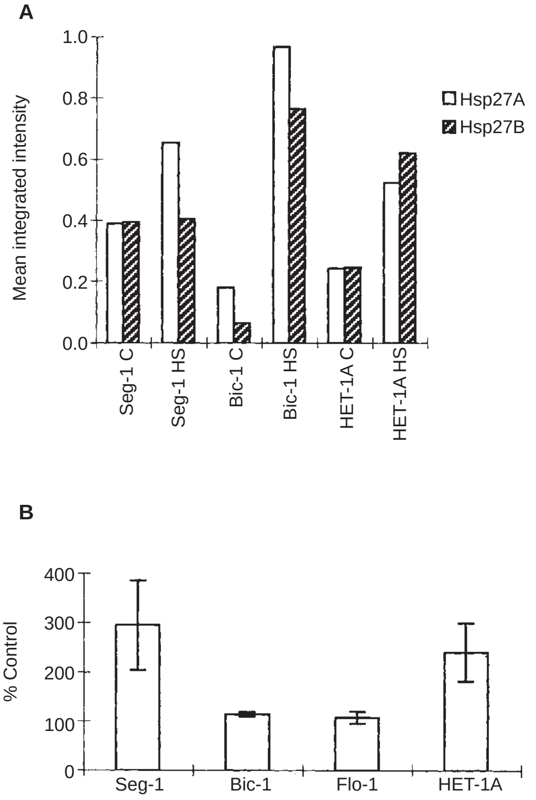

**Figure 5** Response of cell lines to heat shock. (**A**) The Barrett's adenocarcinoma cell lines (Seg-1, Bic-1) and oesophageal epithelial cell line (HET-1A) were heat shocked at 42°C for 2 h and then incubated for 24 h at 37°C. The untreated controls (**C**) and the heat shocked cell lines (HS) were analysed by 2D-PAGE for changes in Hsp27A and B isoform expression. (**B**) Northern analysis of Hsp27 mRNA expression after heat shock. The Barrett's adenocarcinoma cell lines (Seg-1, Bic-1, Flo-1) and HET-1A were heat shocked at 42°C for 2 h and then incubated at 37°C for 16 h. The mean increase over untreated controls in Hsp27 mRNA expression from triplicate experiments is shown; bars, s.e.

were examined. Hsp27 mRNA levels in the normal oesophageal tissue of ten men and ten women with the diagnosis of squamous cell oesophageal cancer were compared. The normal oesophagus from these patients was chosen to minimize the potential effects of chronic gastro-oesophageal reflux on Hsp27 mRNA in patients with Barrett's adenocarcinoma. A significantly higher mean normalized Hsp27 mRNA level was observed in the normal oesophagus of women (4.1-fold higher) than in the men ($P < 0.02$) (Figure 4A). Rehybridization with a cDNA for GSTπ demonstrated no difference between the sexes, suggesting that the difference is specific to Hsp27. To examine whether possible differences in Hsp27 expression may result from the potentially Hsp27-inducing effects of chronic reflux, we compared the normalized Hsp27 mRNA levels in the normal oesophagus of nine women with squamous cell carcinomas and nine women with Barrett's adenocarcinomas (Figure 4B). The mean mRNA levels were 4.0 vs. 4.4, which was not statistically different ($P = 0.82$).

Expression of Hsp27 protein was also assessed in the normal oesophagus specimens of the same ten men and ten women with squamous cell carcinoma examined by Northern analysis. Because quantitative comparisons of Hsp27 in the oesophageal squamous mucosa using techniques that homogenize tissues may be affected by expression within other cell types, such as the muscle cells of the muscularis mucosa, we chose to utilize immunohistochemistry. This technique allows comparison of Hsp27 expression just in the squamous epithelium. The slides were reviewed by two examiners, blinded to the identity and sex of the patients. The slides were rated as to the intensity of staining of the squamous epithelium on a scale of 0 to 3. Negative controls were all graded as 0. The mean staining intensity scores of men were 2.4 for one examiner and 2.1 for the second examiner. The mean scores for the women were 2.2 and 1.7 for the two examiners. The difference in scores was not statistically significant ($P > 0.51$ and $P > 0.24$), indicating similar Hsp27 protein levels in the normal oesophagus of men and women.

Western blot analysis of the total protein extract detected only one 27-kDa Hsp27 protein specific band in the oesophageal samples, confirming a high degree of specificity of the antibody for this protein (data not shown).

### Induction of Hsp27 by heat shock

2D-PAGE was used to examine whether Hsp27 protein levels are increased in response to heat shock in two Barrett's adenocarcinoma cell lines and in the immortalized oesophageal epithelial cell line HET-1A. Seg-1, Bic-1 and HET-1A cells were heat shocked for 2 h at 42°C and then incubated for 24 h at 37°C before collection. The untreated control cultures were incubated for 26 h at 37°C. All three cell lines displayed a heat-shock response, with increases in the mean integrated spot intensities for Hsp27A and B (Figure 5A). The heat-shock response was also examined by Northern analysis. The oesophageal cell lines were heat shocked for 2 h at 42°C in triplicate experiments. An increase in the mean normalized Hsp27 mRNA level with heat shock was observed in the three adenocarcinoma cell lines (Seg-1, Bic-1, Flo-1) and in HET-1A, but this increase did not achieve statistical significance (Figure 5B).

### Adenocarcinoma and epithelial cell lines do not respond to oestradiol treatment

The response of the adenocarcinoma cell line Seg-1 and the oesophageal epithelial cell line HET-1A to 17β-oestradiol was

 © Cancer Research Campaign 1999

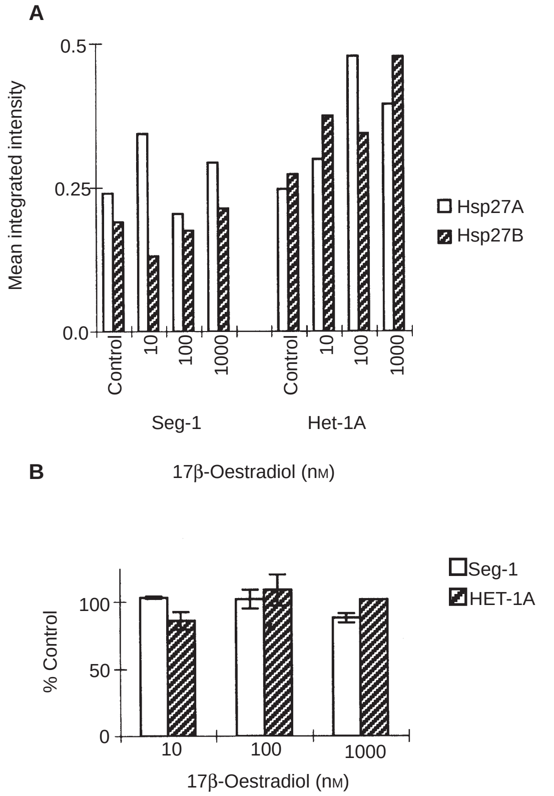

**Figure 6** Effect of 17β-oestradiol on Hsp27 protein and mRNA in Seg-1 and HET-1A cell lines. The cell lines were incubated with 10, 100 and 1000 nM 17β-oestradiol in DMEM and 10% FBS for 48 h at 37°C. (**A**) A small, inconsistent response to oestradiol was observed by 2D-PAGE. (**B**) Northern blot analysis of Hsp27 mRNA expression in 17β-oestradiol-treated Seg-1 and HET-1A cells after normalization using the 28S rRNA probe. Results are expressed as a percentage of the untreated control. Experiments were performed in duplicate (except HET-1A at 1000 nM in which only one trial was performed); bars, s.e.

investigated using both 2D-PAGE and Northern analysis. Seg-1 and HET-1A were chosen because they appeared to have the greatest mean transcriptional response to heat shock. Eighty per cent confluent plates of Seg-1 and HET-1A were each incubated at either 10, 100 or 1000 nM 17β-oestradiol for 48 h and collected for 2D-PAGE analysis. Only a small, and inconsistent response to oestradiol was observed (Figure 6A). Northern analysis showed no consistent transcriptional response of Hsp27 to 17β-oestradiol (Figure 6B).

### DISCUSSION

Multiple studies have demonstrated the protective effect of Hsp27 protein expression against a wide variety of cytotoxic stresses (Landry et al, 1989; Mehlen et al, 1995*a*, 1995*b*; Hout et al, 1991, 1996; Richards et al, 1996; Wu and Welsh, 1996). The abundance of Hsp27 in the oesophageal stratified squamous epithelium suggests that it may be one cellular mechanism by which the oesophagus is protected against certain cytotoxic insults. The normal oesophageal squamous epithelium is replaced by a glandular columnar epithelium known as Barrett's metaplasia after chronic gastro-oesophageal reflux (Barrett, 1950, 1957). 2D-PAGE studies revealed that Hsp27 protein is highly abundant in the normal oesophagus, but only very low levels are detectable in Barrett's metaplasia and Barrett's-associated adenocarcinomas. One consequence of the development of Barrett's metaplasia is that a tissue with abundant Hsp27 expression is replaced by a metaplastic epithelium with very low levels of cytoprotective Hsp27 protein. Our examination of the polypeptide patterns on the 2D-PAGE gels with respect to other heat-shock proteins revealed that they were not abundantly expressed in the oesophageal tissues (data not shown). Thus, Hsp27 expression in normal oesophagus is unique relative to other heat-shock proteins.

Barrett's metaplasia is a premalignant lesion associated with an increased risk of adenocarcinoma (Spechler et al, 1984; Van Der Veen et al, 1989). It arises in the setting of long-standing chronic gastro-oesophageal reflux and its attendant oesophagitis and mucosal injury. The distal oesophagus in patients with acute and chronic reflux oesophagitis is often infiltrated with a variety of inflammatory cells. The lamina propria of Barrett's metaplasia may have varying degrees of acute and chronic inflammation (Hamilton et al, 1992). A causal relationship between chronic inflammation and some malignancies has been proposed (Trush and Kensler, 1991; Ames et al, 1993). The inflammatory response results in the production of reactive oxygen species (ROS) by infiltrating activated macrophages and neutrophils. Furthermore, there is evidence to suggest that reflux oesophagitis results in oxidative stress in the oesophageal mucosa (Wetscher et al, 1995*a–c*). Elevated mucosal ROS concentrations have been demonstrated by chemiluminescence in mucosal biopsy specimens from patients with macroscopic oesophagitis and from patients with Barrett's metaplasia with or without oesophagitis (Olayee et al, 1995). Free radicals injure cell membranes and are mutagenic (Hinder and Stein, 1991; McBride et al, 1991). Huot et al (1996) demonstrated that overexpression of Hsp27 provides protection against actin disruption and cell death by oxyradicals. Similarly, Mehlen et al (1995*b*) demonstrated that expression of small heat-shock proteins protects against cell death induced by TNF-α, hydrogen peroxide or menadione. They reported that small heat-shock proteins decreased the level of intracellular ROS, increased the intracellular level of the antioxidant glutathione and abolished the burst of intracellular ROS induced by TNF-α (Mehlen et al, 1996), an inflammatory cytokine produced by activated macrophages (Fiers, 1991). Low expression of Hsp27 in Barrett's metaplastic columnar epithelium may render this tissue more sensitive to the mutagenic effects of chronic exposure to oxyradicals released or induced by inflammatory cells, leading to neoplastic progression.

It is well established that chronic reflux in the distal oesophagus leads to chronic inflammation, severe oxidative stress and potentially the development of Barrett's metaplasia. We suggest that the high levels of Hsp27 in the normal squamous epithelium may be one protective mechanism against cell death and mutagenesis induced by oxyradicals produced as a consequence of acute and chronic inflammation. If the reflux is chronic and severe, however, the squamous epithelium may be denuded and replaced by an abnormal metaplastic glandular epithelium that is able to tolerate the direct cytotoxic effect of gastro-oesophageal reflux, perhaps because of mucus production. However, this tissue is low in Hsp27, a stress-response protein that reduces intracellular ROS. Barrett's metaplasia and fundic gastric tissue fail to engage the Hsp27 induction pathway in response to chronic reflux. The reflux-induced inflammation and oxidative stress may exert a chronic mutagenic effect on the metaplastic epithelium, leading to dysplasia and carcinogenesis.

© Cancer Research Campaign 1999

The pattern of Hsp27 protein in the oesophagus using immunohistochemistry revealed intense expression localized to the stratified squamous epithelium. Much less Hsp27 was present in the mitotic basal layers, but it was present at high levels in the upper layers of the squamous mucosa. The pattern of staining suggests that the level of Hsp27 may increase with differentiation of the oesophageal epithelium. Hsp27 has previously been implicated as a marker of differentiation. In the cervical epithelium, and in the keratinocytes of normal squamous epidermis and epidermal neoplasms, Hsp27 expression correlates with the degree of cellular differentiation (Dressler et al, 1986; Trautinger et al, 1995). Weak or no expression of the protein correlates with epidermal malignancy (Trautinger et al, 1995). Barrett's metaplasia, Barrett's adenocarcinomas, cardia tumours and gastric tissue all either stained weakly or were negative for Hsp27. In contrast, squamous cell carcinomas displayed high levels of Hsp27 staining and intermediate levels of Hsp27 by 2D-PAGE. These findings are consistent with the origin of these tumours from the squamous epithelium, which strongly expresses Hsp27.

To investigate potential mechanisms which may explain the high levels of Hsp27 in the oesophageal mucosa, but low levels in Barrett's mucosa, Southern and Northern blot analyses were performed. No deletions or major alterations in Hsp27 were observed in Barrett's metaplasia or adenocarcinoma. Results of Northern analysis correlated well with the 2D-PAGE and immunohistochemistry data, indicating that Hsp27 is regulated at the level of mRNA expression. Expression of Hsp27 mRNA was significantly lower in Barrett's metaplasia, Barrett's adenocarcinomas, cardia tumours and gastric tissue compared with normal oesophagus.

Both Barrett's metaplasia and oesophageal adenocarcinoma are more common in men (Skinner et al, 1983; Cameron et al, 1985; Reed and Johnson, 1993), although the biological basis for the increased susceptibility is unknown. One possible explanation may be related to relatively more abundant or effective protective mechanisms in the oesophagus of women which limits damage leading to metaplasia and neoplasia. Hsp27 protein levels are regulated by oestrogen in some breast carcinoma cell lines (Fuqua et al, 1989) and the target organs for oestrogen, such as the female reproductive tract (oviduct, vagina, uterus), are observed to express Hsp27 protein (Ciocca et al, 1983). Dunn et al (1993) have shown a general, but not absolute, correlation between oestrogen receptor positivity and the abundance of Hsp27 protein in a variety of cell lines. To investigate potential gender-related differences in oesophageal Hsp27, we compared the normal oesophageal mucosa of men and women patients by Northern analysis and immunohistochemistry. Hsp27 mRNA expression was 4.1-fold greater in women ($P < 0.02$), but this difference was not evident at the protein level by immunohistochemical analysis. This suggests that either the quantitative differences in Hsp27 mRNA may not result in increased Hsp27 protein or that the immunohistochemical analyses may not be sufficiently quantifiable to detect this difference.

Pharmacological manipulation of Hsp27 levels may be a method by which neoplastic progression of Barrett's metaplasia could be diminished. Because Hsp27 protein expression is stimulated by heat shock and is regulated by oestrogen in some cell lines, we examined the effects of these stimuli. There was an increase in the mean Hsp27 mRNA level in three adenocarcinoma cell lines (Seg-1, Bic-1, Flo-1) and the immortalized oesophageal epithelial cell line (HET-1A) with heat shock, but this did not achieve statistical significance. However, little, if any, effect of 17β-oestradiol treatment was observed in Seg-1 and HET-1A cells either in the level of Hsp27 mRNA or protein. Further studies will examine the effect of oestradiol and other compounds on the phosphorylation state of this protein. The results in the present studies suggest that oestrogen may not be the sole factor underlying the increased expression of Hsp27 in the female oesophagus. This result is consistent with the higher levels of Hsp27 mRNA in women because most were elderly and, therefore, postmenopausal, and none received oestrogen replacement therapy. Therefore, oestrogen therapy may not be useful in increasing expression of Hsp27 protein. Further studies are needed to identify agents which increase Hsp27 expression in columnar epithelium, establish the potential mechanisms for the sex-related differences, and elucidate the protective role of Hsp27 in the oesophagus.

### ACKNOWLEDGEMENTS

We are grateful to Dr Yoshihiro Nambu, Robert Hinderer and Keith Compton for technical assistance; Dr Robert Gilmont for providing pHS 208 plasmid; and Drs Gary Stoner and Paul Hollenberg for the providing HET-1A cell line. This work was supported by American Cancer Society grant CN-171 (DGB) and NIH Training grant CA09672-06 (OSS, SJH).

### REFERENCES

Al-Kasspooles M, Moore JH, Orringer MB and Beer DG (1993) Amplification and overexpression of the *EGF*R and *erb*B-2 genes in human esophageal adenocarcinomas *Int J Cancer* **54**: 213–219

Ames BN, Shigenaga MK and Hagen TM (1993) Oxidants, antioxidants, and the degenerative diseases of aging. *Proc Natl Acad Sci, USA*. **90**: 7915–7922

Arrigo A-P and Welch WJ (1987) Characterization and purification of the small 28,000-Dalton mammalian heat shock protein. *J Biol Chem* **262**: 15359–15369

Barrett NR (1950) Chronic peptic ulcer of the oesophagus and 'oesophagitis'. *Br J Surg* **38**: 175–182

Barrett NR (1957) The lower esophagus lined by columnar epithelium. *Surgery* **41**: 881–894

Blot WJ, Devesa SS, Kneller RW and Fraumeni JF (1991) Rising incidence of adenocarcinoma of the esophagus and gastric cardia. *JAMA* **265**: 1287–1289

Bremner CG, Lynch VP, and Ellis FH (1970) Barrett's esophagus: congenital or acquired? An experimental study of esophageal mucosal regeneration in the dog. *Surgery* **68**: 209–216

Cameron AJ, Ott BJ and Payne WS (1985) The incidence of adenocarcinoma in columnar-lined (Barrett's) esophagus. *N Engl J Med* **313**: 857–859

Ciocca RD, Adams DJ, Edwards DP, Bjercke RJ and McGuire WL (1983) Distribution of an estrogen-induced protein with a molecular weight of 24,000 in normal and malignant human tissues and cells. *Cancer Res* **43**: 1204–1210

Dressler LG, Ramzy I, Sledge GW and McGuire WL (1986) A new marker of maturation in the cervix: the estrogen-regulated 24k protein. *Obstet Gynecol* **68**: 825–831

Dunn DK, Whelan RDH, Hill B and King RJB (1993) Relationship of HSP27 and oestrogen receptor in hormone sensitive and insensitive cell lines. *J Steroid Biochem Mol Biol* **46**: 469–479

Edwards DP, Adams DJ, Savage N and McGuire WL (1980) Estrogen induced synthesis of specific proteins in human breast cancer cells. *Biochem Biophys Res Commun* **93**: 804–812

Fiers W (1991) Tumor necrosis factor: characterization at the molecular, cellular and in vivo level. *FEBS Lett* **285**: 199–212

Fuqua SAW, Blum-Salingaros M and McGuire WL (1989) Induction of the estrogen-regulated '24K' protein by heat shock. *Cancer Res* **49**: 4126–4129

Goldman MC and Beckman RC (1960) Barrett syndrome: case report with discussion about concepts of pathogenesis. *Gastroenterology* **39**: 104–110

Hamilton SR (1992) Oesophagitis. In *Pathology of the Gastrointestinal Tract*. Ming S-C and Goldman H (eds), pp. 381–438. WB Saunders: Philadelphia

Hanash SM, Baier LJ, McCurry L and Schwartz SA (1986) Lineage-related polypeptide markers in acute lymphoblastic leukemia detected by two-dimensional gel electrophoresis. *Proc Natl Acad Sci USA* **83**: 807–811

© Cancer Research Campaign 1999

Hanash SM, Kuick R, Nichols D and Stoolman L (1988) Quantitative analysis of a new marker for common acute lymphoblastic leukemia detected by two-dimensional electrophoresis. *Dis Markers* **6**: 209–220

Hanson LA, Nuzum EO, Jones BC, Malkinson AM and Beer DG (1991) Expression of the glucocorticoid receptor and K-*ras* genes in urethan-induced mouse lung tumors and transformed cell lines. *Exp Lung Res* **17**: 371–387

Hesketh PJ, Clapp RW, Doos WG and Spechler SJ (1989) The increasing frequency of adenocarcinoma of the esophagus. *Cancer* **64**: 526–530

Hickey E, Brandon SE, Sadis S, Smale G and Weber LA (1986) Molecular cloning of sequences encoding the human heat-shock proteins and their expression during hyperthermia. *Gene* **43**: 147–154

Hinder RA and Stein HJ (1991) Oxygen-derived free radicals. *Arch Surg* **126**: 104–105

Hout J, Roy G, Lambert H, Chretien P and Landry J (1991) Increased survival after treatments with anticancer agents of Chinese hamster cells expressing the human $M_r$ 27,000 heat shock protein. *Cancer Res* **51**: 5245–5252

Hout J, Houle F, Spitz DR and Landry J (1996) Hsp27 phosphorylation-mediated resistance against actin fragmentation and cell death induced by oxidative stress. *Cancer Res* **56**: 273–279

Kuick RD, Hanash SM, Chu EHY and Strahler JR (1987) A comparison of some adjustment techniques for use with quantitative spot data from two-dimensional gels. *Electrophoresis* **8**: 199–204

Landry J, Chretien P, Lambert H, Hickey E and Weber LA (1989) Heat shock resistance conferred by expression of the human hsp27 gene in rodent cells. *J Cell Biol* **109**: 7–15

Landry J, Chretien P, Laszlo A and Lambert H (1991) Phosphorylation of Hsp27 during development and decay of thermotolerance in Chinese hamster cells. *J Cell Physiol* **147**: 93–101

Landry J, Lambert H, Zhou M, Lavoie JN, Hickey E, Weber LA and Anderson CW (1992) Human Hsp27 is phosphorylated at serines 78 and 82 by heat shock and mitogen-activated kinases that recognize the same amino acid motif as S6 kinase II. *J Biol Chem* **267**: 794–803

Lavoie JN, Lambert H, Hickey E, Weber LA and Landry J (1995) Modulation of cellular thermoresistance and actin filament stability accompanies phosphorylation-induced changes in the oligomeric structure of heat shock protein 27. *Mol Cell Biol* **15**: 505–516

McBride TJ, Preston BD and Loeb LA (1991) Mutagenic spectrum resulting from DNA damage by oxygen radicals. *Biochemistry* **30**: 207–213

Mehlen P, Mehlen A, Guillet D, Preville X and Arrigo A-P (1995*a*) Tumor necrosis factor-α induces changes in the phosphorylation, cellular localization, and oligomerization of human hsp27, a stress protein that confers cellular resistance to this cytokine. *J Cell Biochem* **58**: 248–259

Mehlen P, Preville X, Chareyron P, Briolay J, Klemenz R and Arrigo A-P (1995*b*) Constitutive expression of human hsp27, *Drosophila* hsp27, or human aB-crystallin confers resistance to TNF- and oxidative stress-induced cytotoxicity in stably transfected murine L929 fibroblasts. *J Immunol* **154**: 363–374

Mehlen P, Kretz-Remy C, Preville X and Arrigo A-P (1996) Human hsp27, *Drosophila* hsp27 and human αB-crystallin expression-mediated increase in glutathione is essential for the protective activity of these proteins against TNFα-induced cell death. *EMBO J* **15**: 2695–2706

Moscow JA, Fairchild CR, Madden MJ, Ransom DT, Wieand HS, O'Brien EE, Poplack DG, Cossman J, Myers CE and Cowan KH (1989) Expression of anionic glutathione-S-transferase and p-glycoprotein genes in human tissues and tumors. *Cancer Res* **49**: 1422–1428

Naef AP, Savary M and Ozzello L (1975) Columnar-lined lower esophagus: an acquired lesion with malignant predisposition. *J Thorac Cardiovasc Surg* **70**: 826–835

Olyaee M, Sontag S, Salman W, Schnell T, Mobarhan S, Eiznhamer D and Keshavarzian A (1995) Mucosal reactive oxygen species production in oesophagitis and Barrett's oesophagus. *Gut* **37**: 168–173

Rachwal WJ, Bongiorno PF, Orringer MB, Whyte RI, Ethier SP and Beer DG (1995) Expression and activation of erbB-2 and epidermal growth factor receptor in lung adenocarcinomas. *Br J Cancer* **72**: 56–64

Reed PI and Johnson BJ (1993) The changing incidence of oesophageal cancer. *Endoscopy* **25**: 606–608

Richards EH, Hickey E, Weber L and Masters JRW (1996) Effect of overexpression of the small heat shock protein HSP27 on the heat and drug sensitivities of human testis tumor cells. *Cancer Res* **56**: 2446–2451

Schnell T, Sontag S, Wanner J, Chintam R, Chejfec G, O'Connell S and Moroni B (1985) Endoscopic screening for Barrett's esophagus (BE), esophageal adenocarcinoma (AdCa) and other mucosal changes in ambulatory subjects with symptomatic gastroesophageal reflux (GER). *Gastroenterology* **88**: 1576

Skinner DB, Walther BC, Riddell RH, Schmidt H, Iascone C and Demeester TR (1983) Barrett's esophagus. Comparison of benign and malignant cases. *Ann Surg* **198**: 554–566

Spechler SJ, Robbins AH, Rubins HB, Vincent ME, Heeren T, Doos WG, Colton T and Schimmel EM (1984) Adenocarcinoma and Barrett's esophagus: an overrated risk? *Gastroenterology* **87**: 927–933

Stoner GD, Kaighn ME, Reddel RR, Resau JH, Bowman D, Naito Z, Matsukura N, You M, Galati AJ and Harris CC (1991) Establishment and characterization of SV40 T-antigen immortalized human esophageal epithelia cells. *Cancer Res* **51**: 365–371

Strahler JR, Kuick R, Eckerskorn C, Lottspeich F, Richardson BC, Fox DA, Stoolman LM, Hanson CA, Nichols D, Tueche HJ and Hanash SM (1990) Identification of two related markers for common acute lymphoblastic leukemia as heat shock proteins. *J Clin Invest* **85**: 200–207

Trautinger F, Kindas-Mugge I, Dekrout B, Knobler RM and Metze D (1995) Expression of the 27-kDa heat shock protein in human epidermis and in epidermal neoplasms: an immunohistological study. *Br J Dermatol* **133**: 194–202

Trush MA and Kensler TW (1991) An overview of the relationship between oxidative stress and chemical carcinogenesis. *Free Radical Biol Med* **10**: 201–209

Van Der Veen AH, Dees J, Blankensteijn JD and Van Blankenstein M (1989) Adenocarcinoma in Barrett's oesophagus: an overrated risk. *Gut* **30**: 14–18

Welch WJ (1985) Phorbol ester, calcium ionophore, or serum added to quiescent rat embryo fibroblast cells all result in the elevated phosphorylation of two 28,000-Dalton mammalian stress proteins. *J Biol Chem* **260**: 3058–3062

Wetscher GJ, Hinder PR, Bagchi D, Perdikis G, Redmond EJ, Glaser K, Adrian TE and Hinder RA (1995*a*) Free radical scavengers prevent reflux esophagitis in rats. *Dig Dis Sci* **40**: 1292–1296

Wetscher GJ, Hinder RA, Bagchi D, Hinder PR, Bagchi M, Perdikis G and McGinn T (1995*b*) Reflux esophagitis in humans is mediated by oxygen-derived free radicals. *Am J Surg* **170**: 552–557

Wetscher GJ, Perdikis G, Kretchmar DH, Stinson RG, Bagchi D, Redmond EJ, Adrian TE and Hinder RA (1995*c*) Esophagitis in Sprague-Dawley rats is mediated by free radicals. *Dig Dis Sci* **40**: 1297–1305

Wheater PR, Burkitt HG and Daniels VG (1987) *Functional Histology: a Text and Colour Atlas*, pp. 64–78. Churchill Livingstone: New York

Winters C, Spurling TJ, Chobanian SJ, Curtis DJ, Esposito RL, Hacker JF, Johnson DA, Cruess DF, Cotelingam JD, Gurney MS and Cattau EL (1987) Barrett's esophagus: a prevalent, occult complication of gastroesophageal reflux disease. *Gastroenterology* **92**: 118–124

Wu W and Welsh MJ (1996) Expression of the 25-kDa heat-shock protein (HSP27) correlates with resistance to the toxicity of cadmium chloride, mercuric chloride, *cis*-platinum(II)-diammine dichloride, or sodium arsenite in mouse embryonic stem cells transfected with sense or antisense HSP27 cDNA. *Toxicol Appl Pharmacol* **141**: 330–339

© Cancer Research Campaign 1999

## References

[PDF_00776] BARRETT N. R. (1950). Chronic peptic ulcer of the oesophagus and 'oesophagitis'.. Br J Surg.

[PDF_00778] BARRETT N. R. (1957). The lower esophagus lined by columnar epithelium.. Surgery.

[PDF_00780] Blot W. J., Devesa S. S., Kneller R. W., Fraumeni J. F. (1991). Rising incidence of adenocarcinoma of the esophagus and gastric cardia.. JAMA.

[PDF_00782] Bremner C. G., Lynch V. P., Ellis F. H. (1970). Barrett's esophagus: congenital or acquired? An experimental study of esophageal mucosal regeneration in the dog.. Surgery.

[PDF_00785] Cameron A. J., Ott B. J., Payne W. S. (1985). The incidence of adenocarcinoma in columnar-lined (Barrett's) esophagus.. N Engl J Med.

[PDF_00787] Ciocca D. R., Adams D. J., Edwards D. P., Bjercke R. J., McGuire W. L. (1983). Distribution of an estrogen-induced protein with a molecular weight of 24,000 in normal and malignant human tissues and cells.. Cancer Res.

[PDF_00790] Dressler L. G., Ramzy I., Sledge G. W., McGuire W. L. (1986). A new marker of maturation in the cervix: the estrogen-regulated 24K protein.. Obstet Gynecol.

[PDF_00793] Dunn D. K., Whelan R. D., Hill B., King R. J. (1993). Relationship of HSP27 and oestrogen receptor in hormone sensitive and insensitive cell lines.. J Steroid Biochem Mol Biol.

[PDF_00796] Edwards D. P., Adams D. J., Savage N., McGuire W. L. (1980). Estrogen induced synthesis of specific proteins in human breast cancer cells.. Biochem Biophys Res Commun.

[PDF_00799] Fiers W. (1991). Tumor necrosis factor. Characterization at the molecular, cellular and in vivo level.. FEBS Lett.

[PDF_00801] Fuqua S. A., Blum-Salingaros M., McGuire W. L. (1989). Induction of the estrogen-regulated "24K" protein by heat shock.. Cancer Res.

[PDF_00803] GOLDMAN M. C., BECKMAN R. C. (1960). Barrett syndrome. Case report with discussion about concepts of pathogenesis.. Gastroenterology.

[PDF_00807] Hanash S. M., Baier L. J., McCurry L., Schwartz S. A. (1986). Lineage-related polypeptide markers in acute lymphoblastic leukemia detected by two-dimensional gel electrophoresis.. Proc Natl Acad Sci U S A.

[PDF_00812] Hanash S. M., Kuick R., Nichols D., Stoolman L. (1988). Quantitative analysis of a new marker for common acute lymphoblastic leukemia detected by two-dimensional electrophoresis.. Dis Markers.

[PDF_00815] Hanson L. A., Nuzum E. O., Jones B. C., Malkinson A. M., Beer D. G. (1991). Expression of the glucocorticoid receptor and K-ras genes in urethan-induced mouse lung tumors and transformed cell lines.. Exp Lung Res.

[PDF_00818] Hesketh P. J., Clapp R. W., Doos W. G., Spechler S. J. (1989). The increasing frequency of adenocarcinoma of the esophagus.. Cancer.

[PDF_00820] Hickey E., Brandon S. E., Sadis S., Smale G., Weber L. A. (1986). Molecular cloning of sequences encoding the human heat-shock proteins and their expression during hyperthermia.. Gene.

[PDF_00823] Hinder R. A., Stein H. J. (1991). Oxygen-derived free radicals.. Arch Surg.

[PDF_00829] Huot J., Houle F., Spitz D. R., Landry J. (1996). HSP27 phosphorylation-mediated resistance against actin fragmentation and cell death induced by oxidative stress.. Cancer Res.

[PDF_00825] Huot J., Roy G., Lambert H., Chrétien P., Landry J. (1991). Increased survival after treatments with anticancer agents of Chinese hamster cells expressing the human Mr 27,000 heat shock protein.. Cancer Res.

[PDF_00841] Landry J., Lambert H., Zhou M., Lavoie J. N., Hickey E., Weber L. A., Anderson C. W. (1992). Human HSP27 is phosphorylated at serines 78 and 82 by heat shock and mitogen-activated kinases that recognize the same amino acid motif as S6 kinase II.. J Biol Chem.

[PDF_00845] Lavoie J. N., Lambert H., Hickey E., Weber L. A., Landry J. (1995). Modulation of cellular thermoresistance and actin filament stability accompanies phosphorylation-induced changes in the oligomeric structure of heat shock protein 27.. Mol Cell Biol.

[PDF_00849] McBride T. J., Preston B. D., Loeb L. A. (1991). Mutagenic spectrum resulting from DNA damage by oxygen radicals.. Biochemistry.

[PDF_00859] Mehlen P., Kretz-Remy C., Préville X., Arrigo A. P. (1996). Human hsp27, Drosophila hsp27 and human alphaB-crystallin expression-mediated increase in glutathione is essential for the protective activity of these proteins against TNFalpha-induced cell death.. EMBO J.

[PDF_00851] Mehlen P., Mehlen A., Guillet D., Preville X., Arrigo A. P. (1995). Tumor necrosis factor-alpha induces changes in the phosphorylation, cellular localization, and oligomerization of human hsp27, a stress protein that confers cellular resistance to this cytokine.. J Cell Biochem.

[PDF_00855] Mehlen P., Preville X., Chareyron P., Briolay J., Klemenz R., Arrigo A. P. (1995). Constitutive expression of human hsp27, Drosophila hsp27, or human alpha B-crystallin confers resistance to TNF- and oxidative stress-induced cytotoxicity in stably transfected murine L929 fibroblasts.. J Immunol.

[PDF_00863] Moscow J. A., Fairchild C. R., Madden M. J., Ransom D. T., Wieand H. S., O'Brien E. E., Poplack D. G., Cossman J., Myers C. E., Cowan K. H. (1989). Expression of anionic glutathione-S-transferase and P-glycoprotein genes in human tissues and tumors.. Cancer Res.

[PDF_00867] Naef A. P., Savary M., Ozzello L. (1975). Columnar-lined lower esophagus: an acquired lesion with malignant predisposition. Report on 140 cases of Barrett's esophagus with 12 adenocarcinomas.. J Thorac Cardiovasc Surg.

[PDF_00870] Olyaee M., Sontag S., Salman W., Schnell T., Mobarhan S., Eiznhamer D., Keshavarzian A. (1995). Mucosal reactive oxygen species production in oesophagitis and Barrett's oesophagus.. Gut.

[PDF_00876] Reed P. I., Johnston B. J. (1993). The changing incidence of oesophageal cancer.. Endoscopy.

[PDF_00878] Richards E. H., Hickey E., Weber L., Master J. R. (1996). Effect of overexpression of the small heat shock protein HSP27 on the heat and drug sensitivities of human testis tumor cells.. Cancer Res.

[PDF_00885] Skinner D. B., Walther B. C., Riddell R. H., Schmidt H., Iascone C., DeMeester T. R. (1983). Barrett's esophagus. Comparison of benign and malignant cases.. Ann Surg.

[PDF_00888] Spechler S. J., Robbins A. H., Rubins H. B., Vincent M. E., Heeren T., Doos W. G., Colton T., Schimmel E. M. (1984). Adenocarcinoma and Barrett's esophagus. An overrated risk?. Gastroenterology.

[PDF_00891] Stoner G. D., Kaighn M. E., Reddel R. R., Resau J. H., Bowman D., Naito Z., Matsukura N., You M., Galati A. J., Harris C. C. (1991). Establishment and characterization of SV40 T-antigen immortalized human esophageal epithelial cells.. Cancer Res.

[PDF_00895] Strahler J. R., Kuick R., Eckerskorn C., Lottspeich F., Richardson B. C., Fox D. A., Stoolman L. M., Hanson C. A., Nichols D., Tueche H. J. (1990). Identification of two related markers for common acute lymphoblastic leukemia as heat shock proteins.. J Clin Invest.

[PDF_00899] Trautinger F., Kindas-Mügge I., Dekrout B., Knobler R. M., Metze D. (1995). Expression of the 27-kDa heat shock protein in human epidermis and in epidermal neoplasms: an immunohistological study.. Br J Dermatol.

[PDF_00903] Trush M. A., Kensler T. W. (1991). An overview of the relationship between oxidative stress and chemical carcinogenesis.. Free Radic Biol Med.

[PDF_00908] Welch W. J. (1985). Phorbol ester, calcium ionophore, or serum added to quiescent rat embryo fibroblast cells all result in the elevated phosphorylation of two 28,000-dalton mammalian stress proteins.. J Biol Chem.

[PDF_00911] Wetscher G. J., Hinder P. R., Bagchi D., Perdikis G., Redmond E. J., Glaser K., Adrian T. E., Hinder R. A. (1995). Free radical scavengers prevent reflux esophagitis in rats.. Dig Dis Sci.

[PDF_00914] Wetscher G. J., Hinder R. A., Bagchi D., Hinder P. R., Bagchi M., Perdikis G., McGinn T. (1995). Reflux esophagitis in humans is mediated by oxygen-derived free radicals.. Am J Surg.

[PDF_00917] Wetscher G. J., Perdikis G., Kretchmar D. H., Stinson R. G., Bagchi D., Redmond E. J., Adrian T. E., Hinder R. A. (1995). Esophagitis in Sprague-Dawley rats is mediated by free radicals.. Dig Dis Sci.

[PDF_00922] Winters C., Spurling T. J., Chobanian S. J., Curtis D. J., Esposito R. L., Hacker J. F., Johnson D. A., Cruess D. F., Cotelingam J. D., Gurney M. S. (1987). Barrett's esophagus. A prevalent, occult complication of gastroesophageal reflux disease.. Gastroenterology.

[PDF_00926] Wu W., Welsh M. J. (1996). Expression of the 25-kDa heat-shock protein (HSP27) correlates with resistance to the toxicity of cadmium chloride, mercuric chloride, cis-platinum(II)-diammine dichloride, or sodium arsenite in mouse embryonic stem cells transfected with sense or antisense HSP27 cDNA.. Toxicol Appl Pharmacol.

